# Effects of Therapeutic and Aerobic Exercise Programs on Pain, Neuromuscular Activation, and Bite Force in Patients with Temporomandibular Disorders

**DOI:** 10.3390/jpm11111170

**Published:** 2021-11-10

**Authors:** Paula Manuela Mendes Moleirinho-Alves, Pedro Miguel Teixeira Cravas Cebola, Paulo Duarte Guia dos Santos, José Pedro Correia, Catarina Godinho, Raul Alexandre Nunes da Silva Oliveira, Pedro Luís Cemacelha Pezarat-Correia

**Affiliations:** 1 CIPER Neuromuscular Research Lab, Faculty of Human Kinetics, University of Lisbon, 1499-002 Cruz Quebrada, Portugal; l2013123@fmh.ulisboa.pt (P.D.G.d.S.); jpcorreia.ft@gmail.com (J.P.C.); roliveira@fmh.ulisboa.pt (R.A.N.d.S.O.); ppezarat@fmh.ulisboa.pt (P.L.C.P.-C.); 2 Grupo de Patologia Médica, Nutrição e Exercício Clínico (PaMNEC) do Centro de Investigação Interdisciplinar Egas Moniz (CiiEM), 2829-511 Monte de Caparica, Portugal; cebola_ped@hotmail.com (P.M.T.C.C.); cgcgodinho@gmail.com (C.G.); 3 Sleep and Temporomandibular Disorder Department, Cuf Tejo Hospital, 130-352 Lisboa, Portugal

**Keywords:** temporomandibular disorders, physiotherapy, surface electromyography, aerobic exercise, therapeutic exercise

## Abstract

Pain in masticatory muscles is one of the most frequent symptoms in patients with temporomandibular disorders (TMD) and can lead to changes in the patterns of neuromuscular activity of masticatory muscles and decrease in bite force. This study assesses the effects of three eight-week exercise programs on pain intensity, neuromuscular activation, and bite force of masticatory muscles in patients with TMD. Forty-five patients were divided into three groups: a therapeutic exercise program (G1), a therapeutic and aerobic exercise program (G2), and an aerobic exercise program (G3). The masticatory muscles’ pain was evaluated using the numeric pain rating scale (NPRS), surface electromyographic (sEMG) activity of the masseter was recorded during maximum voluntary contraction and at rest, and bite force was evaluated using a dynamometer. These parameters were evaluated twice at baseline (A01/A02), at the end of the eight-week intervention period (A1), and 8–12 weeks after the end of the intervention (A2). After intervention, G2 showed the best results, with a significantly decrease in masticatory muscles’ pain and increase in bite force. These results suggest that interventions to reduce pain in patients with TMD should be multimodal.

## 1. Introduction

Temporomandibular disorder (TMD) is the second most common cause of mouth and face pain, only surpassed by odontogenic pain, and presents a high potential to evolve into persistent/chronic pain [1,2]. It is estimated that the worldwide prevalence is between 5% and 12% of the adult population and that women are affected at least twice as much as men [3]. Regarding TMD subgroups, myofascial pain disorders have been found in 45.3% of patients, and women constitute the majority of patients in all TMD subgroups, especially in muscle-related TMD [4]. The most frequent symptoms of TMD muscle-related dysfunction are pain, changes in mouth opening, and jaw movements [1]. However, pain is the main issue and the most common reason for seeking medical care [5], which is why a large number of studies have aimed to evaluate the effectiveness of various pain-related intervention measures [6]. Pain in the masticatory muscles in individuals with TMD can lead to changes in neuromuscular activation patterns, which may be more evident in maximum voluntary contraction (MVC) [7], with some studies indicating a decrease in bite force (BF) in these patients [8].

Several studies carried out using different approaches in TMD have obtained contradictory results [9,10,11,12]. Physiotherapy plays a prominent role in the treatment of TMD [13] and has several intervention strategies, including therapeutic exercises [14,15]; however, although several studies have evaluated the effects of therapeutic exercise on pain control [13,14,15], the significant methodological heterogeneity among studies makes it difficult to reach a consensus. Exercise is an important component of pain management strategies for most patients and is also vital for their general health and well-being [15]. Several studies report that exercise reduces the sensitivity to painful stimuli in healthy individuals [15,16,17,18,19], which is termed exercise-induced analgesia or hypoalgesia [20,21,22]. High-intensity aerobic exercise has been known to produce a large hypoalgesic effect in healthy individuals [23]; other studies carried out in subjects with chronic pain, including fibromyalgia, undergoing aerobic exercise have also shown that intense exercise can decrease pain [24,25]. However, there is a lack of studies investigating the role of aerobic exercise in patients with TMD. Therefore, the present study aims to assess the effects of three eight-week exercise intervention programs on pain, neuromuscular activity, and bite force of masticatory muscles in patients with muscle-related TMD.

## 2. Materials and Methods

### 2.1. Study Design

In the present longitudinal study, a non-probabilistic convenience sampling method was used.

### 2.2. Sampling and Recruitment

Participants were recruited between July 2019 and February 2020 at Egas Moniz University Clinic and Egas Moniz Dental Clinic by two dentists and a physiotherapist, all three being specialists in TMD with over five years’ experience in the field. The inclusion criteria were 18–50 years of age, a diagnosis of local myalgia and myofascial pain (for bilateral masseter and temporalis muscles) according to Diagnostic Criteria for Temporomandibular Disorders (DC-TMD) [26], and signing the free and informed consent form. Patients with any musculoskeletal, psychiatric, cardiovascular, pulmonary, autoimmune, active malignant neoplastic, metabolic, and/or neurological disease; with any other medical consideration that prevented the performance of aerobic exercises of moderate intensity; a history of face trauma; using orthodontic and/or occlusal bite appliances; with pregnancy, alcoholism or drug addiction, drug intake that can affect neuromuscular system, or who had used painkillers and or anti-inflammatory drugs in the 48 h prior to data collection were excluded.

The sample size was calculated using GPower 3.0 version 3.0.10 (Heinrich-Heine-Universität, Dusseldorf, Germany), and a total of 45 subjects were recruited according to the highest value obtained in the different statistical methodologies used and considering an alpha of 5% and a power of 80%.

### 2.3. Ethics and Procedures

The study protocol was approved by the Ethical Committee of Egas Moniz University Institute on 13 February 2019 (ID N° 675/2019). All individuals gave their informed consent in accordance with the Helsinki Declaration and understood that they were free to withdraw from the study at any time.

**Figure 1:**
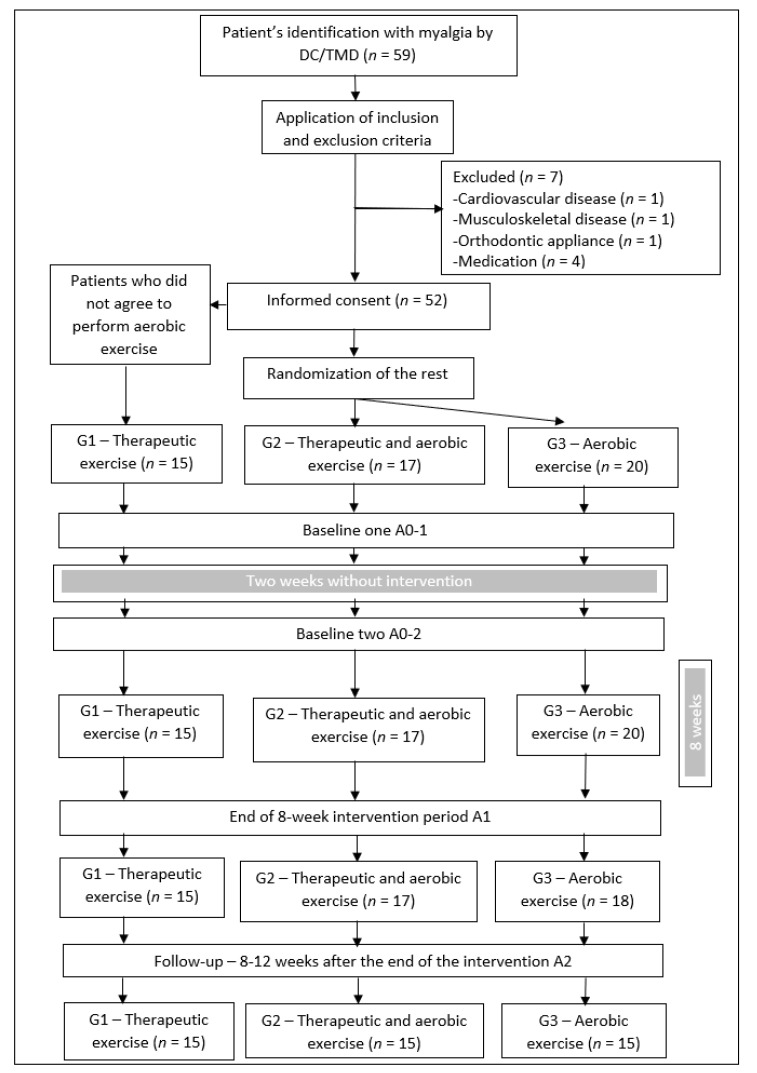
Study design flow chart.

Patients were distributed among the three exercise groups. Those who did not agree to perform aerobic exercise were allocated to experimental group 1 (G1), and the remaining patients were randomly allocated to the other two groups (G2 and G3), using a computer program (randomized.com). G1 carried out a protocol of specific therapeutic exercises for the masticatory muscles, G2 carried out the same therapeutic exercise protocol directed at the masticatory muscles associated with aerobic exercise, and G3 carried out the same aerobic exercise program as G2. At the first moment of assessment (A01), pain intensity and sEMG data at the resting position (RP) and maximum voluntary contraction (MVC) were collected. Two weeks later, a new evaluation (A02) was carried out without any intervention between these two moments. Then, the G1, G2, and G3 exercise programs were started. Eight weeks after the start of the intervention protocols, a new evaluation moment (A1) was carried out, which took place 48 h after the end of the exercise programs. Eight to twelve weeks after the end of the intervention, the patients returned to the clinic for the last evaluation moment (A2) (Figure 1). All evaluation data were collected by an independent researcher who was blinded to the group allocation.

Patients in the G1 group participated in a weekly exercise session for a period of 8 weeks. The physiotherapy session was the same for all participants, the techniques were always applied in the same sequence, and they were always performed by the same physiotherapist for 30 min. Each session consisted of the following techniques: compression, transverse, and longitudinal massage of the masseter muscle, bilaterally; longitudinal massage of the temporal muscle, bilaterally; compression of the medial pterygoid muscle, bilaterally; passive stretching of the masseter and medial pterygoid, bilaterally; isotonic strengthening exercises through resisted mouth opening and closing and resisted left and right deviation (10 repetitions of each exercise); and coordination exercises through mouth opening and closing exercises and laterals (10 repetitions of each exercise) [27,28,29,30].

Patients in the G2 group participated in a weekly physiotherapy session for a period of 8 weeks. The physical therapy session was the same as described for G1. The G2 group exercise program also included two weekly cycle ergometer training sessions, which were always supervised by the same physiotherapist and lasted for 30 min. The participants cycled the first 5 min (warm-up period) with an intensity of 50% of the heart rate reserve (HRR), the next 24 min with an intensity of 70% of the HRR, and the last minute at 50% of the HRR for active recovery. The speed and/or resistance of the cycle ergometer were adjusted throughout the training period in order to keep the exercise intensity within the predefined value. HRR was determined according to the Karvonen formula [31], and resting heart rate (HR) was assessed on three consecutive days, after five minutes of rest in a chair with the arms supported. The average value was calculated and used as the resting HR.

Patients in the G3 group underwent only two weekly cycle ergometer training sessions for eight weeks, which were always supervised by the same physiotherapist and lasted for 30 min. The protocol performed was the same as that defined for G2.

### 2.4. Data Collection

Pain intensity was evaluated using an analog algometer (Baseline^®^ 11LBS model, 5 kg, with 1 cm [2] of stainless steel contact surface, Fabrication Enterprises Inc., White Plains, NY, USA), with the patients comfortably positioned in the supine position and fully relaxed. The evaluator placed the end of the contact surface of the algometer perpendicularly and exerted a gradual pressure on the three portions of the masseter (upper, middle, and lower) and temporal (anterior, middle, and posterior) muscles bilaterally, according to the physical examination indications of the DC-TMD [26]. Changes in pain intensity were evaluated by applying a constant pressure (1 Kg) [26] using the NPRS. The subjects were instructed to classify the pain intensity at the maximum pressure exerted. Pain was measured three times at each of the points mentioned above, with an interval of 10 s between each measure at the same point and 30 s between different points. For each point, the average of the three measurements was calculated to determine the NPRS. The minimum clinically important difference considered was two points [32].

sEMG procedures and data acquisition were performed by a physiotherapist with previous experience in sEMG. All procedures and sEMG data were collected with patients in a calm and quiet environment with a constant room temperature, seated comfortably in a chair without head support, hands resting on their legs and aligned with their shoulders, hips, and knees at 90° of flexion, and instructed to look ahead and avoid facial and orbicular expressions. Before the start of data collection, sEMG data acquisition procedures were prepared. AMBU^®^ BlueSensor N electrodes (AMBU, Ballerup, Denmark), which were bipolar (pre-gelled Ag-AgCl, 10 mm diameter disc), were positioned parallel to the muscle fibers and fixed on the skin in the masseter muscle (superficial portion), bilaterally. To determine electrode placement, an isometric contraction of the masseter in maximum intercuspation was requested, in order to guarantee the same position of the temporomandibular joint was maintained in all subsequent procedures. The electrodes were placed in the muscle belly, with the upper electrode at the intersection between the tragus-labial commissure and the exocanthion–gonion lines [33], and were positioned 20 mm apart according to SENIAM recommendations [34]. Skin preparation was performed by gentle sanding and cleaning with cotton wool soaked in a 70% alcoholic solution. A bracelet ground electrode was placed over the wrist styloid apophyses. For the collection of sEMG data, an eight-channel system with active electrodes connected to a bioPLUX research 2010 system (PLUX, Lisbon, Portugal) was used, with a common mode rejection ratio of 110 dB, input impedance > 100 MΩ, and gain of 1000. The data were recorded with a sampling frequency of 1000 Hz. Prior to processing, the raw sEMG signals were inspected by an experienced researcher to assess their quality. Afterwards, the signals were digitally filtered (10–490 Hz), rectified, and smoothed using a 4th-order 5-Hz Butterworth low-pass filter. The normalization of the resting sEMG value was performed using the MVC sEMG value as a reference. To quantify sEMG intensity, the average EMG signal amplitude was determined during the defined time periods. All sEMG signal processing was performed through routines developed with Matlab software (The Mathworks Inc., Natick, MA, USA). The recording of sEMG activity in the RP was performed by maintaining the masticatory muscles at rest for one minute. The 10 s between 40 and 50 s were selected to assess muscle activity at rest. sEMG during MVC was assessed by maintaining a maximum contraction for three seconds and was controlled using a bite force dynamometer (model IDDK, Kratos Equipamentos Industriais Ltd.a, Cotia, São Paulo, Brazil), which was placed between the first and second premolars. The defined procedure was performed bilaterally and repeated three times on each side. Both used software programs, sEMG, and Matlab, were configured to store all generated data files in a solid state disk encrypted with Bitlocker, accessible only to the principal investigator of this study. All collected data were properly anonymized.

### 2.5. Data Analysis

Categorical variables were analyzed using Pearson’s Chi-Square Test to confirm equality between groups at the time A01. For continuous variables, a non-parametric Kruskal–Wallis Test was performed to confirm equality between groups at time A01. A two-way mixed ANOVA was used to assess the behavior of the variables at the various assessment times (random factor) and for the three intervention groups (fixed factor). A 95% confidence interval was determined for all tests, and a 5% significance level was used. The Statistical Package for the Social Sciences (SPSS^®^) version 26 (IBM Corp., Armonk, NY, USA) was used for all statistical analyses. Cohen’s d effect size was also calculated: effect sizes up to 0.2 were considered irrelevant, those between 0.2 and 0.5 were considered small, those between 0.5 and 0.8 were considered moderate, and values above 0.8 were considered large.

## 3. Results

The sample consisted of 52 patients, out of which 45 (86.5%) completed the four evaluation moments (15 in each of the three experimental groups). Of the 52 initial patients, there were seven dropouts (two who did not attend the third evaluation moment (A-1) and five who did not attend the last evaluation moment (A-2)), which represented a value of 13.5% that confirmed a good adherence to the study. The statistical analysis performed only included the 45 participants who completed the four evaluation moments. Each group was composed of 13 females and 2 males (H = 1.61, *p* = 0.45), ranging from 18 to 35 years of age. The mean age and standard deviation was 26.9 ± 5.5 years for G1, 26 ± 4.4 years for G2, and 24.9 ± 3.4 years for G3. In G1 and G2, myofascial pain was the most common diagnostic subgroup (86.7%), while myalgia (86.7%) was the most common in G3. The distribution of diagnostic subgroups was significantly different between the exercise groups (χ^2^ = 22.88, *p* < 0.001).

## 4. Pain Intensity

**Figure 2:**
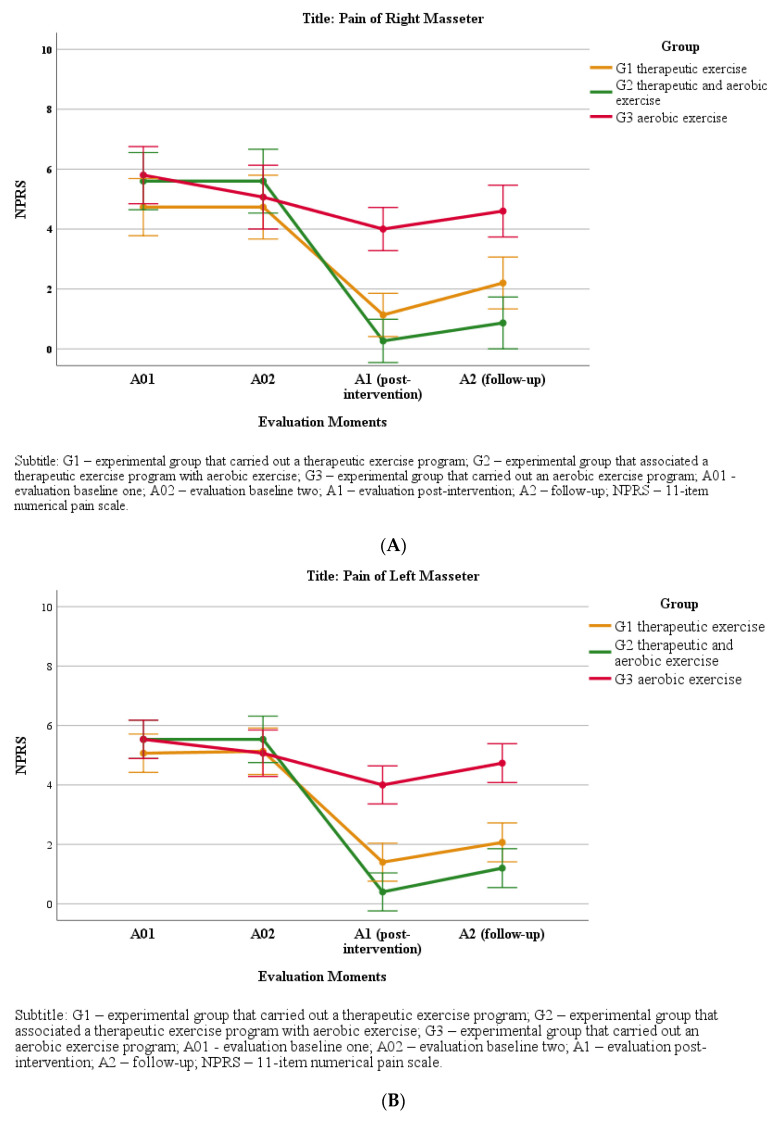

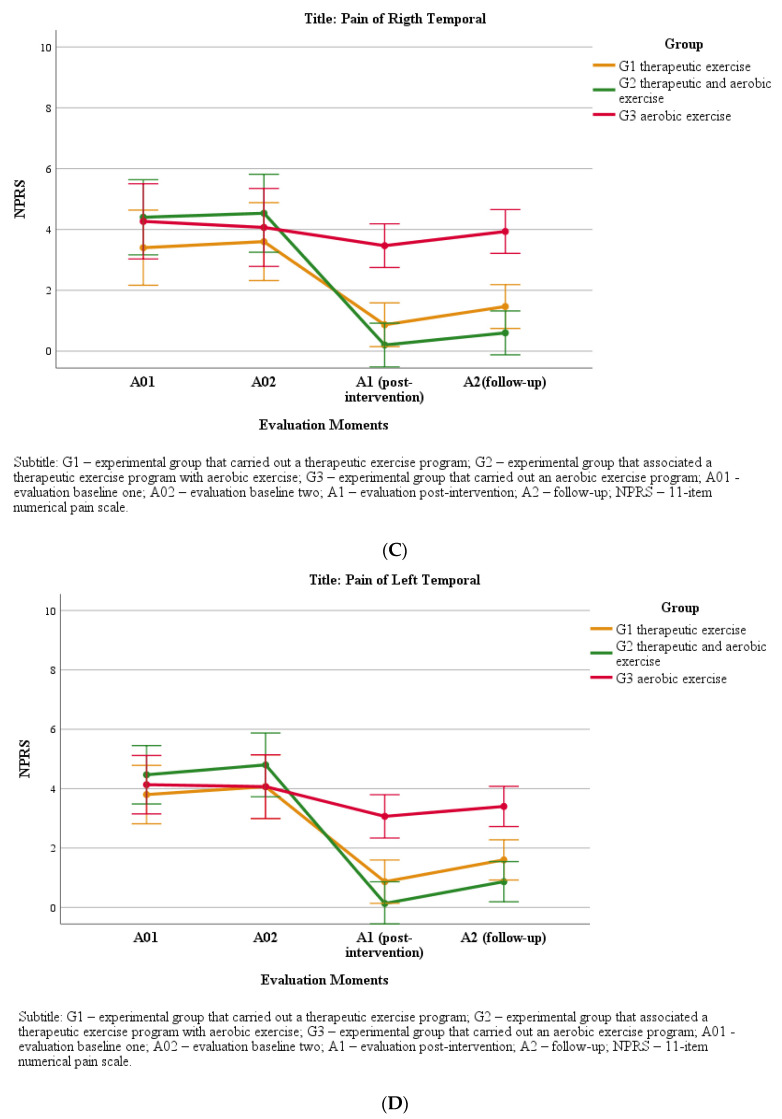
(**A**) Changes in the NPRS of the right masseter muscle at the different time points. (**B**) Changes in the NPRS of the left masseter muscle at the different time points. (**C**) Changes in the NPRS of the right temporal muscle at the different time points. (**D**) Changes in the NPRS of the left temporal muscle at the different time points.

Both the left and right masseter and temporalis muscles’ NPRS did not present significant differences (H = 2.1, *p* = 0.36; H = 0.30, *p* = 0.86 for the left and right masseter, respectively; H = 0.54, *p* = 0.77; H = 0.18, *p* = 0.91 for the left and right temporalis, respectively) between groups at either A01 or A02. After the intervention programs (A1), NPRS improved significantly more in G2 and G1 than in G3. The effect size of this difference was large for the masseter muscle in the three groups; however, the G2 group stood out for this indicator, with d = 4.0 on the right and 5.4 on the left masseter, respectively; in the other groups, there were effect sizes of 2.1 and 2.8 in G1 (right and left sides, respectively) and of 1.0 and 1.3 in G3 (right and left sides, respectively). Regarding the temporal muscle, there was a large effect on G1, with values of 1.2 and 1.8 (right and left sides, respectively) and on G2, with 2.3 and 3.3 (right and left sides, respectively), while G2 showed moderate effect sizes of d = 0.4 and 0.5 in the right and left sides, respectively. Between A1 and A2, there was a marginal increase in the mean of the NPRS in all groups, which was neither significant nor had a notable effect size (Figure 2A–D).

## 5. Neuromuscular Activity—sEMG

The average amplitude of the normalized sEMG signal was calculated for masseter muscle of both sides (left and right). In resting position, the average sEMG was calculated for a time window of 10 s (between 40 and 50 s) and in the MVC for a time window of 0.250 s around the maximum peak.

There were no significant differences between groups in the mean sEMG of the masseter muscle during MVC (H = 4.98, *p* = 0.19; H = 0.18, *p* = 0.91 for the left and right sides, respectively) or RP (H = 0.71, *p* = 0.54; H = 1.25, *p* = 0.92 for the left and right sides, respectively) on either side at A01 or A02. There were also no significant differences in MVC and RP sEMG between groups at A1, A2, or between these two time points (Table 1).

## 6. Bite Force

**Figure 3:**
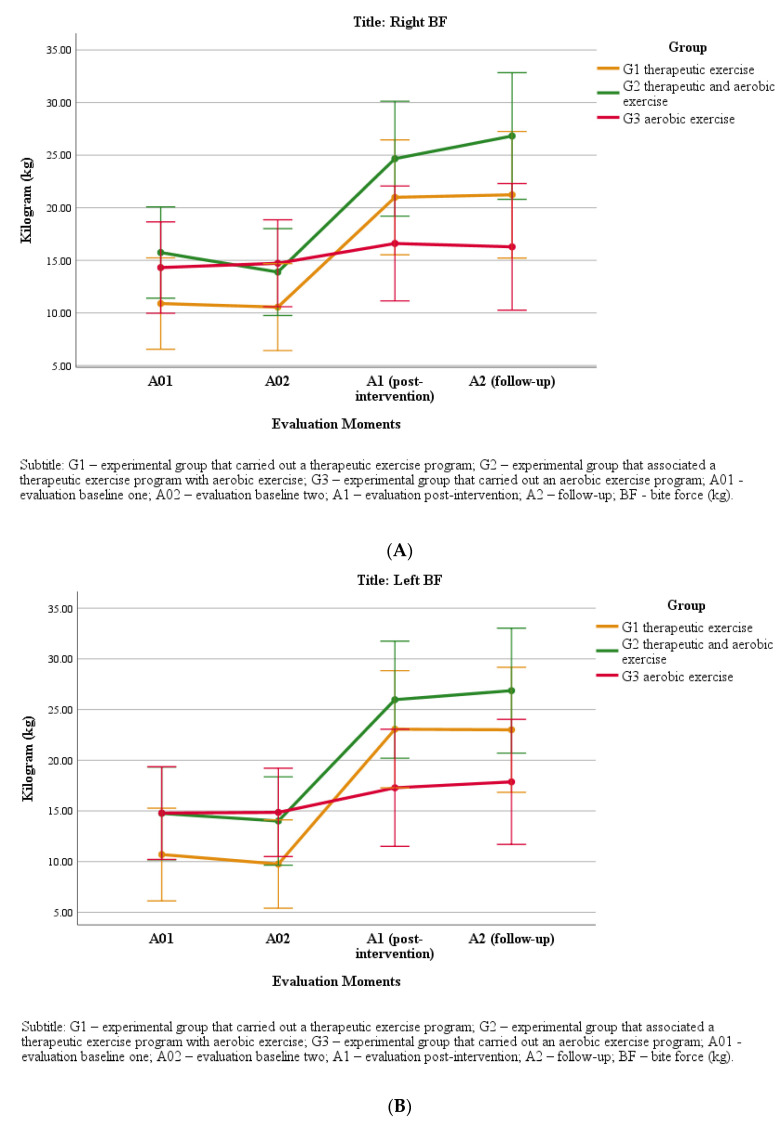
(**A**) Changes in right BF at the different time points. (**B**) Changes in left BF at the different time points.

There were no differences in BF (H = 1.25, *p* = 0.54; H = 2.31, *p* = 0.31 for the left and right sides, respectively) between groups either on the right or the left sides at both A01 and A02. From baseline to A1, BF significantly increased in G1 and G2, while G3 showed no significant differences. There was a large effect size in G1 on the right (d = 1.5) and left (d = 1.7) sides and on the left side in G2 (d = 0.8), while a moderate effect size was seen in the G2 group on the right side (d = 0.7), and a small effect size was seen in G3 both on the right and on the left sides (d = 0.3). There were no significant changes from A1 to A2 on either side in any of the groups (Figure 3A,B).

## 7. Discussion

To our knowledge, this is the first study to analyze the effects of a combination of therapeutic and aerobic exercise on participants with TMD. A treatment regimen of aerobic exercise combined with therapeutic exercise over an 8-week period significantly decreased pain and increased BF. However, changes in sEMG activity were not significant. The study hypothesis was therefore partially confirmed.

## 8. Pain Intensity

Pain decreased in all groups after the intervention programs were carried out, with a greater effect size for the G2 group, followed by G1. Changes in pain were evaluated by applying a constant pressure (1 Kg), using the numerical pain scale (NPRS). The minimum clinically important difference considered was two points [32].

In G1 and G2, the intervention programs led to a clinically important reduction in NPRS, with this reduction being greater in G2. In G3, there was no clinically significant reduction in NPRS after performing the aerobic exercise program.

NPRS was higher for the masseter muscle (right and left) than for the temporal muscle (right and left) in all groups. This difference between muscles may result from the average of the initially reported NPRS, which was always higher for the masseter muscle, since when analyzing the mean NPRS values after the eight-week intervention period, the reported final NPRS values were similar for both muscles (masseter and temporal) bilaterally.

The results obtained in G1 are in line with those observed by Kalamir et al. [35], who found that a therapeutic exercise program led to a reduction in pain in the masticatory muscles. The different type of intervention (which included intra-oral myofascial therapy, condylar distraction, isometric contractions, and mobilization of the temporal and medial pterygoid) and duration (two weekly sessions for five weeks) relative to our study does not allow for further comparisons.

In G3, the results obtained in the masseter and temporal muscle were in line with those identified by Naugle et al. [16], in which the authors concluded that aerobic exercise led to a wide range of hypoalgesic effects (between 0.04 and 1.47). However, it is necessary to interpret these data carefully, since the cited study was carried out in healthy individuals.

Studies of aerobic exercise in patients with chronic pain, including fibromyalgia, have found a decrease in pain with a subset of physical activity [24,25]. Since patients with TMD show central sensitization [36,37], the aerobic exercise seem to promote similar results and should be included in TMD intervention. Thus, the results obtained by G3, in relation to the masseter (large effect size) and the temporal (moderate effect size) muscle, may result from the majority of the participants in this group having myalgia (presence of local pain on palpation) and as such having less change in pain processing than patients with health conditions that lead to major changes in central pain processing, such as those involving the presence of myofascial pain (radiating pain on palpation within the limits of the assessed muscle). Patients with myofascial pain can present an increase in the size of receptor fields, a greater susceptibility to painful stimuli, and a lower threshold of neuronal depolarization. These differences may be due to the different subtypes of TMD, with some patients presenting only peripheral involvement and others presenting both peripheral and central involvement [38].

The group with better results (greater effect sizes) was the one that associated therapeutic exercises with aerobic exercises (G2), which suggests that the simultaneous use of both types of intervention improves outcomes. However, it is important to note that the effects seen in G1 showed a pattern of effects similar to those observed in G2, but smaller. The larger effect sizes seen in G2 may result from the association between the effect of therapeutic exercise and the decrease in sensitivity to painful stimuli as a result of the activation of opioid and non-opioid systems resulting from aerobic exercise. Exercise induces the release of endogenous opioids in peripheral, spinal, and/or central sites, which leads to pain modulation [39] and can also contribute to the release of neurotransmitters (serotonin and norepinephrine) according to the defined exercise parameters [40], which can produce analgesia when activating cannabinoid receptors [22]. Additionally, the increase in blood pressure and heart rate during exercise [41], as well as the activation of descending pathways [42], may have contributed to hypoalgesia. Considering the multidimensional experience of pain and the complexity of the processes involved in the mechanisms of analgesia, the most likely explanation is that there may be an interaction between different systems, in different patients, and at different times depending on the set of factors that in each situation are contributing to the presence of pain.

The effects obtained immediately after the completion of the intervention programs were maintained during the final follow-up in all groups.

## 9. Bite Force and Neuromuscular Activation of Masticatory Muscles

The sEMG activity during MVC did not change significantly in any of the groups after the intervention programs. Previous studies that carried out intervention strategies to decrease pain in masticatory muscles also did not find changes in the masseter sEMG activity after the decrease in pain intensity [43,44]. However, comparisons are difficult to make, since in those studies the normalization of MVC occurred through the interposition of folded articulating paper between the teeth, and the authors did not use a dynamometer to control the BF, as was done in the present study.

A study of experimentally induced masseter pain found that the increase in pain led to a decrease in sEMG activity, not only during the period when the pain was present, but also after its disappearance [45]. The fact that most of the patients in our study reported pain in the masticatory muscles for more than six months may have contributed to the lack of variation in sEMG activity, since it may already be the result of an anticipatory and protective response developed by the central nervous system (CNS). In the final follow-up, there were also no changes in sEMG activity in any of the groups. Thus, in our study, there was no change in the sEMG activity of patients after the intervention programs were performed, despite the decrease in pain intensity seen in all groups.

BF increased in all groups after the completion of the intervention programs, with greater effect sizes in G1 and G2, while the increase was much lower in G3.

The results are in line with those observed by other authors, who found that the decrease in masticatory muscle pain intensity led to an increase in BF [46]. Another set of studies evaluating the BF of patients with TMD in comparison with healthy subjects found that the masticatory MVC was reduced in the group of participants with TMD [8,47]. Cho and Lee [7] obtained similar results, since they found that the presence of pain led to a decrease in BF, although it is necessary to emphasize that in these studies, pain was experimentally induced. There remains no consensus on the relationship between pain in the masticatory muscles and BF, since other authors have not identified this correlation [33,48]. In the present study, the group with better results (as demonstrated by the greater effect size) was G1, followed by G2. There seems to be a relationship between the decrease in pain in the masticatory muscles and the increase in BF, since in G1 and G2, where there was a greater decrease in pain intensity, a greater increase in BF was also observed.

The different results observed for relationship between BF and pain in different studies [7,33,35] may be due to the patients presenting different levels of pain intensity, anxiety, fear, pain catastrophizing, and different psychological characteristics [49]. The sensory system, one of the components of the neuromuscular system, is multidimensional and includes several inter-individual factors, with contributions from sensory-discriminatory, cognitive-evaluative and motivational-affective components, where factors such as the pain location, intensity, and characteristics can lead to supraspinal and suprabulbar modulation, thus modifying the effects of pain on motor activity [48,50].

Several hypotheses can be theorized to explain the increase in BF without a corresponding increasing in sEMG activity during MVC. Given the complexity of the neuromuscular system, the increased BF without increasing MVC sEMG may result from the development of different combinations of muscle activation strategies, which may show intra- and inter-individual variability. For example, a better synchronization between muscles can be a strategy to coordinate muscle activity and lead to increased force production without changes in the activation intensity in each muscle [51]. The increase in strength may also have resulted from an increase in synergistic muscle activity regardless of activation neural activity of agonists [52]. Finally, there may have been a decrease in antagonist co-activation, since it is counterproductive to the production of maximum force because it generates a force opposite to the desired movement [53]. This decreased co-activation of antagonistic muscles would be expected through reciprocal inhibition [54]. On the other hand, sEMG activity depends on the ability of the CNS to direct a central drive to the motoneuron pool, which may be influenced by the pain intensity, levels of anxiety, individual psychological characteristics, fear of movement, and pain catastrophizing [49], which contributes to increasing the complexity of the mechanisms involved. It is plausible to state that the complexity and multidimensionality inherent to the neuromuscular system can lead to different neuromuscular recruitment strategies for the performance of a given task, which are composed by complex and individualized reactions that can lead to an increase in BF without being accompanied by an increase in the sEMG activity during the MVC.

Regarding sEMG in the PR, despite the decreasing trend seen in the three groups after the exercise interventions, there were no significant differences. Studies that have compared the sEMG activity at rest between patients with TMD and healthy individuals have shown contradictory findings, with some authors identifying greater activity in individuals with TMD [55] and others not reporting significant differences [56]. The effects obtained immediately after completing the intervention program were maintained at the final follow-up.

We recommend that physiotherapists carry out multimodal training programs that include therapeutic exercise associated with aerobic exercise to reduce the pain intensity of the masseter and temporal muscles and increase BF in patients with TMD.

This study has some limitations. First, we used a small, convenience-based sample, which means that the findings may not be generalizable to other populations. The sample also included only subjects between 18 and 35 years, which does not allow extrapolation of results to other age groups. The non-acceptance by several patients of joining groups G2 and G3, which included the performance of the aerobic exercise program, may have contributed to some heterogeneity between groups at baseline regarding the type of TMD (in group G3, the majority of patients presented myalgia-type TMD, while in the other groups, the dominant was myofascial-type TMD), but the statistical analysis did not show differences at baseline between the G1 and G2/G3 groups (the differences are between G3 and G1/G2), so the allocation to the groups was considered to have not interfered with the results obtained. Despite the strict compliance with the electrode placement protocol, it is impossible to guarantee that we were able to exactly reproduce the electrode placement in the different evaluations of each subject. In a similar way, despite our efforts to reproduce the dynamometer placement protocol between the premolars in the different evaluations, we cannot be sure that there was no variability in this placement, which might have influenced the BF produced.

Future studies should recruit a larger sample and randomize patients across the three groups so that the results can be extrapolated to other populations. An intervention program which includes aerobic exercises of moderate intensity to facilitate the participants’ adherence and carry out an intervention program with a shorter duration (six weeks) should also be considered to check whether similar results would be obtained in a shorter period of time of intervention.

## 10. Conclusions

The therapeutic exercise program associated with aerobic exercise showed the greatest effects on pain intensity (decrease) and BF (increase), although there were no significant differences in the group that performed therapeutic exercise alone. Interventions in patients with TMD experiencing pain must have a multimodal component, and aerobic exercise was shown to be an important component. There were no changes in the sEMG activity during MVC and at rest after carrying out any of the three exercise programs. All effects obtained after carrying out the exercise programs were maintained at follow-up.

## Figures and Tables

**Table 1:** Changes in sEMG during MVC and RP in each group at the different time points.

		G1 (Mean)	Lower Bound	Upper Bound	G2 (Mean)	Lower Bound	Upper Bound	G3 (Mean)	Lower Bound	Upper Bound
sEMG of MVC and RP			95% Confidence Interval			95% Confidence Interval			95% Confidence Interval	
A01	sEMG Right MVC	93.3	91.672	95.005	92.5	90.858	94.192	92.4	90.736	94.069
	sEMG Left MVC	90.8	89.246	92.265	91.4	89.924	92.943	91.2	89.663	92.682
	Right RP	6.1	4.031	8.044	5.0	2.950	6.963	5.4	3.355	7.367
	Left RP	7.2	4.920	9.560	5.5	3.154	7.794	6.2	3.881	8.521
A02	sEMG Right MVC	90.8	88.938	92.583	91.9	90.068	93.713	90.8	88.978	92.623
	sEMG Left MVC	90.5	88.844	92.161	90.6	88.901	92.218	89.5	87.834	91.151
	Right RP	6.0	3.892	8.144	5.1	3.015	7.267	5.6	3.478	7.730
	Left RP	7.3	4.967	9.553	5.6	3.352	7.938	6.3	3.971	8.557
A1	sEMG Right MVC	92.0	90.353	93.732	92.3	90.570	93.949	92.3	90.653	94.032
	sEMG Left MVC	90.8	88.986	92.668	91.8	89.977	93.658	90.5	88.666	92.347
	Right RP	4.4	2.740	6.115	3.2	1.469	4.844	4.6	2.900	6.276
	Left RP	4.6	2.821	6.385	3.6	1.788	5.352	5.3	3.481	7.045
A2	sEMG Right MVC	92.3	90.584	93.916	92.1	90.445	93.778	91.9	90.221	93.553
	sEMG Left MVC	90.9	89.056	92.667	91.3	89.474	93.084	91.7	89.921	93.531
	Right RP	4.6	2.800	6.431	3.5	1.660	5.292	5.1	3.315	6.947
	Left RP	4.7	2.979	6.560	3.7	1.902	5.484	5.8	4.068	7.650

Abbreviations: G1—experimental group that carried out a therapeutic exercise program; G2—experimental group that associated a therapeutic exercise program with aerobic exercise; G3—experimental group that carried out an aerobic exercise program; A01—evaluation baseline one; A02—evaluation baseline two; A1—evaluation post-intervention; A2—follow-up; sEMG—electromyography activity; MVC—maximal voluntary contraction (%); RP—rest position.

## Data Availability

The data presented in this study are available on request from the first author.
